# Temperature Dependence of the Band Gap and Exciton Photoreflectance in Layered Gallium Telluride

**DOI:** 10.1021/acsami.4c17178

**Published:** 2025-02-03

**Authors:** Carlo C. Sta. Maria, Po-Hung Wu, Denny Pratama Hasibuan, Clara Sinta Saragih, Hien Giap, Duc Huy Nguyen, Yan-Ruei Chen, Giang Thi Phan, Duy Van Pham, Ji-Lin Shen, Chien-Chih Lai, Maw-Kuen Wu, Yuan-Ron Ma

**Affiliations:** ‡ Department of Physics, National Dong Hwa University, Hualien 97401, Taiwan; § Department of Electrical Engineering, National Dong Hwa University, Hualien 97401, Taiwan; ⊥ Department of Physics, Chung Yuan Christian University, Taoyuan 32023, Taiwan; ∥ Institute of Physics, Academia Sinica, Taipei 11529, Taiwan; ¶ Office of Postgraduate Studies, UCSI University, Kuala Lumpur 56000, Malaysia; △ Department of Applied Informatics, Fo Guang University, Yilan 262307, Taiwan

**Keywords:** layered gallium telluride, photoreflectance, conduction band edge transition, free exciton, photodetection

## Abstract

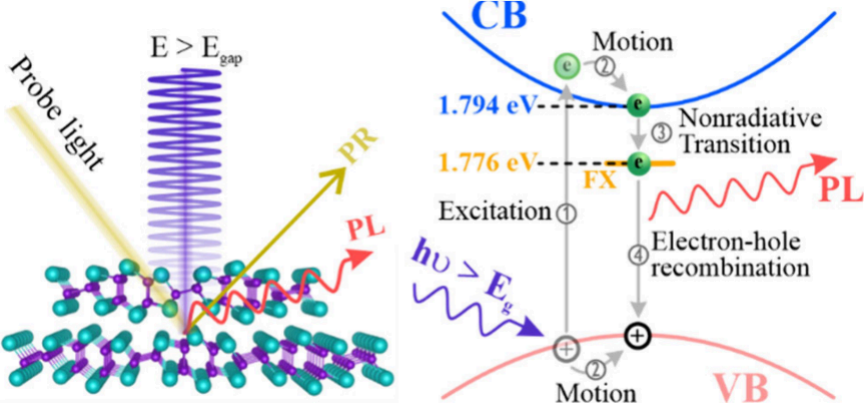

Among Group III-A metal monochalcogenides, gallium telluride (GaTe) is one of the less studied materials in terms of applications and optical characterization. For the temperature dependence of the energy transitions in GaTe, photoluminescence (PL) spectroscopy is commonly used, and photomodulated reflectance (PR) is yet to be reported. In this work, layered monoclinic GaTe single crystals were synthesized by the Bridgman technique and used for the investigation of the conduction band (CB) edge and free-exciton (FX) state transitions using PR spectroscopy. Both energy transitions (i.e., absorption and emission) were present at room temperature at 1.656 and 1.647 eV for the CB edge transition (≡*E*_g_) and for the FX state transition, respectively, and show a blueshift at cryogenic temperatures that can be fitted with Varshni’s equation. The estimated *E*_(0)_ is 1.794 eV for *E*_g_ and 1.776 eV for the FX transitions at 0 K. The energy of the FX state transition is ∼18 meV lower than that of the band gap (*E*_g_) at 0 K. PL spectroscopy confirms that the PL emission is only the FX state transition that is lower than *E*_g_. The temperature-induced band-gap shifting is related to performing temperature-dependent photodetector experiments using various incident light wavelengths. At 80 K, the responsivity of the single-crystal GaTe photodetector to the energies of wavelengths (735 and 845 nm) smaller than *E*_g_ is relatively smaller than that to 630 nm incident light. This indicates that the low-temperature band-gap shift plays a role in applications of GaTe in optoelectronics.

## 1 Introduction

The exceptional properties of layered Group III-A metal monochalcogenides^1,2^ establish them as one of the most extensively studied classes of two-dimensional (2D) materials among others.^3−7^ Optoelectronic devices based on such materials, including field-effect transistors^8^ and photodetectors,^9,10^ demonstrate excellent performance that can match or exceed transition-metal chalcogenides.^11−13^ In addition, several Group III-A metal monochalcogenides are direct-band-gap semiconductors, which is crucial in applications requiring luminescence, such as light-emitting diodes (LEDs)^14^ and solid-state lasers.^10,15,16^ For example, the photoluminescence (PL) emission of gallium monochalcogenides depends on the chalcogen component, such as 2.5 eV for gallium sulfide (GaS),^17^ 2.0 eV for gallium selenide (GaSe),^17,18^ and 1.7 eV for gallium telluride (GaTe).^19,20^ The 2D-layered indium selenide (InSe), on the other hand, has a conduction band (CB) edge emission from 1.25 eV in bulk up to 2.11 eV in a monolayer.^21^ In comparison with other gallium chalcogenides such as GaS and GaSe, GaTe has fewer experimental papers on its optoelectronic properties. Recent reports on GaTe focus on optical anisotropy measurements^20,22^ and low-temperature PL emission studies,^22−24^ which give information on its CB edge excitons. This is important in determining its applications where the band gap plays a role, including photodetectors,^25−32^ photovoltaic devices,^33^ and visible-light photocatalysts for the hydrogen evolution reaction^34^ and degradation of methyl blue.^35^ GaTe exists in monoclinic and hexagonal phases, which give rise to PL emissions at ∼1.65^19,22−24,36,37^ and ∼1.58 eV,^24^ respectively.

Photomodulated reflectance (PR) spectroscopy is a powerful noncontact technique that probes direct energy transitions in semiconductors, including excitons, trions, or emissions from defect states.^38,39^ By normalization of the PR with unmodulated reflectance, any background noise is significantly reduced, leading to an enhancement in energy transition features.^38^ As a result, PR spectroscopy can be more sensitive to free exciton (FX) or other energy transitions compared with other optical spectroscopies. It can be used as a complement to PL spectroscopy by confirming the band-gap energy through a different mechanism. Another type of spectroscopy, thermoreflectance (TR), relies on periodic heating to modulate the lattice, and it has been demonstrated to be consistent with temperature-dependent PL emission studies; however, it is only sensitive to the FX energy transition in multilayer GaTe.^22,24,40^ PR spectroscopy is a versatile method in the characterization of semiconductor materials, yet it is rarely applied in recent optical studies of layered-type semiconductors.^41−43^ Detailed studies on the temperature dependence of PR spectroscopy for layered GaTe are still lacking.^44^ Hence, a comprehensive temperature-dependent PR measurement will be beneficial for the energy transition investigations and applications of GaTe in optoelectronic devices.

In this work, a layered GaTe single-crystal boule was synthesized in an evacuated quartz tube using the Bridgman method. This technique facilitates the growth of a GaTe single crystal consisting of layers held together by van der Waals force. Temperature-dependent Raman spectroscopy was carried out for the as-synthesized GaTe flakes to confirm whether any structural transitions or oxidations are taking place at low temperatures. The optical band gap and FX of GaTe were investigated using PR spectroscopy from room temperature to ∼10 K. Low-temperature PL measurements were used to support the PR spectroscopy by verifying the FX emission through a different method. Last, the effect of temperature on the practical applications of GaTe was demonstrated by conducting cryogenic photodetection experiments, highlighting the impact of temperature-induced band-gap energy shifting.

## 2 Methods

### 2.1 Growth and Chemical Analysis of the Layered GaTe

Layered GaTe ingot was grown using the Bridgman method, as previously reported.^45^ Pure gallium and tellurium powders in a 1:1 molecular ratio were sealed in an evacuated quartz tube at a pressure of ∼10^–5^ Torr. The gallium and tellurium powder mixture was first melted into an alloy at 850 °C and then slowly cooled to ∼20 °C. Next, the quartz tube was placed in a vertical heating furnace that has a temperature gradient of 45 °C/cm from the heating zone to the growth zone. Starting from the heating zone, the sealed quartz tube was lowered at a rate of ∼0.69 cm/day while being rotated at 15 cycles/min. The process takes approximately 9 days, and the resulting ingot consists of van der Waals layered GaTe. Optical microscopy (OM), transmission electron microscopy (TEM), and field-emission scanning electron microscopy (SEM) were used to observe the surface morphology and the layered structure of the single-crystal GaTe. To confirm the elemental composition, the layered GaTe single crystals were analyzed using energy-dispersive X-ray spectroscopy (EDX). Raman spectroscopy studies were performed using a laser confocal microscope with a linearly polarized 633 nm laser and a CCD detector. Low-temperature Raman measurements were done in a vacuum cryostat with a temperature range from room temperature (300 K) to 80 K. Because GaTe degrades when exposed to ambient-air environments,^20,36^ scotch tape was used to peel off the exposed surface layers before the experiments were performed.

### 2.2 Temperature-Dependent Band-Gap Energy Studies of the Layered GaTe

The band-gap energy of the layered GaTe was investigated using PR and PL spectroscopies. The spectroscopic light (“probe”) source was a tungsten–halogen lamp equipped with a monochromator, and the modulation (“pump”) source was a linearly polarized 405 nm continuous-wave laser. An optical chopper (200 Hz) paired with a lock-in amplifier was used to separate the modulated and unmodulated reflectance signals. PR measurements were carried out at cryogenic temperatures (10–300 K) in an ultrahigh-vacuum chamber with a pressure of ∼10^–7^ Torr. In addition to PR spectroscopy, the FX emission was also studied by using low-temperature PL spectroscopy. PL emission measurements were carried out using the same laser confocal microscope mentioned in the previous section (Section 2.1) with a 405 nm laser.

### 2.3 Temperature-Dependent Photodetection of the Layered GaTe

Photodetection experiments were carried out using a multimeter (Keithley 2410 sourcemeter) and a cryogenic stage equipped with a temperature controller (Lakeshore 335 cryogenic temperature controller). A GaTe flake was used as the photoactive sensing material, and glass substrates coated with indium–tin oxide (ITO) were used as the electrical contacts. A simple device consisting of an ITO/glass-GaTe-ITO/glass sandwich configuration was constructed, and LEDs with peak wavelengths of 630, 735, and 845 nm were used as the incident light sources. Photoresponse characterizations were performed inside a vacuum chamber at a pressure of ∼10^–5^ Torr at a temperature range from 300 to 80 K.

## 3 Results and Discussion

### 3.1 Chemical and Structural Characterizations of GaTe

**Figure 1:**
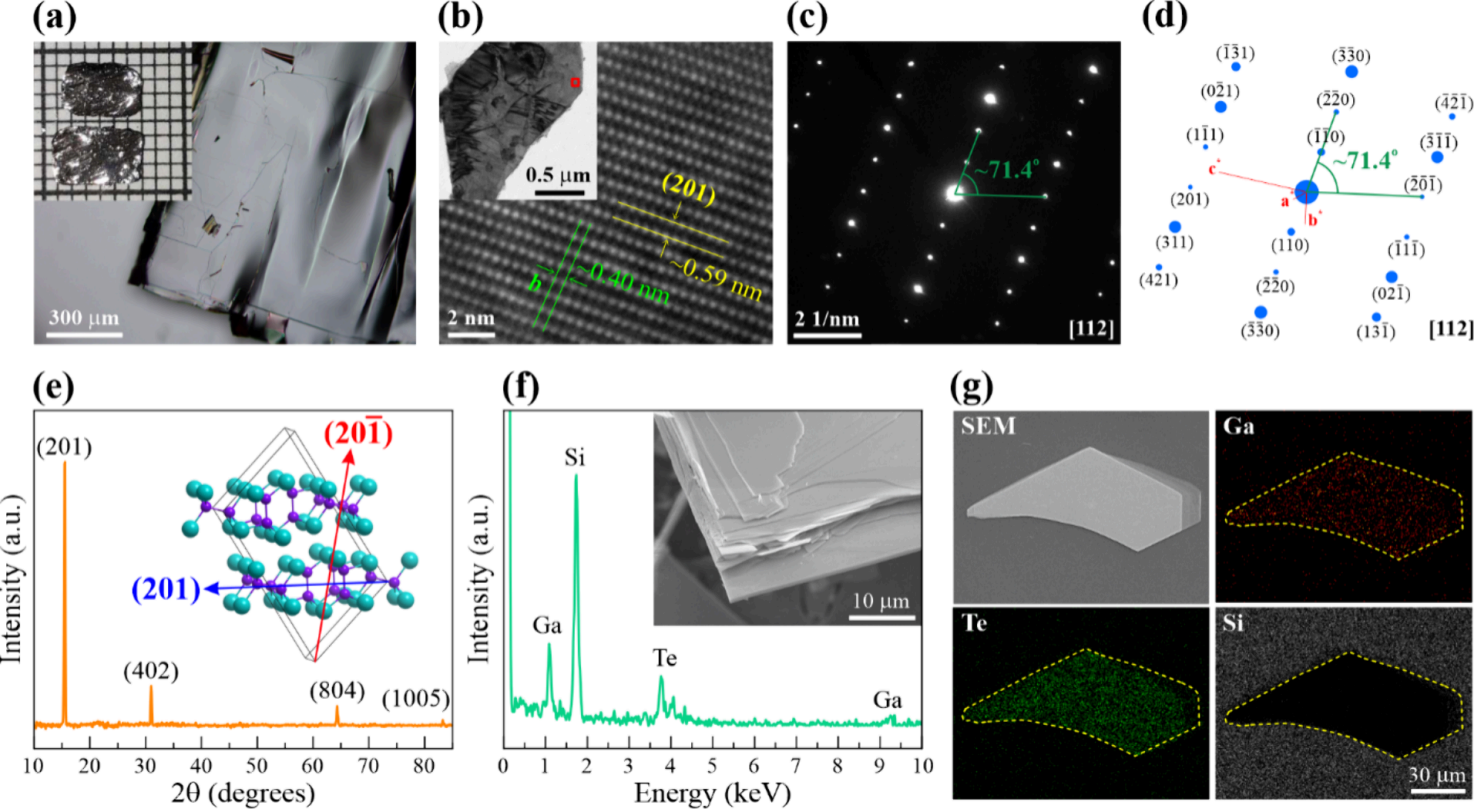
Characterization of the GaTe single crystal. (a) OM image of a large-area single-crystal GaTe flake. Inset: Photograph of an as-synthesized piece that was cut from the single-crystal boule. The grid scale is 1 mm × 1 mm. (b) High-magnification HRTEM image showing (201) interplanar spacing. Inset: Low-magnification image of a GaTe flake on a TEM grid. The highlighted region is the approximate location of the high-magnification HRTEM image. (c) SAED pattern at the [1̅12] zone axis. (d) SAED simulation of monoclinic GaTe. (e) XRD pattern of GaTe indicating a monoclinic crystal structure. (f) EDX spectrum of a GaTe flake. Inset: SEM image highlighting the layered structure of GaTe. (g) EDX mapping of a GaTe flake on silicon.

The physical morphology, crystal structure, and elemental analysis of the single-crystal GaTe flakes are displayed in Figure 1. The single-crystal GaTe flake has a smooth and large-area surface, as shown in the OM image in Figure 1a. The inset shows a photograph of a large GaTe piece, which was cut from the as-synthesized single-crystal boule. TEM was carried out to study the crystalline structure of GaTe. Analysis of the high-magnification high-resolution TEM (HRTEM) image in Figure 1b reveals interplanar spacings of ∼0.59 and ∼0.40 nm, which correspond to the separation of the (201) and *b* planes of monoclinic GaTe, respectively. The high-magnification HRTEM was obtained from the GaTe flake on a carbon film grid shown in the inset. The selected-area electron diffraction (SAED) pattern of the same flake at the [112] zone axis is shown in Figure 1c. The sharp, bright diffraction spots confirm that the as-synthesized GaTe consists of monoclinic single crystals. All diffraction spots from the SAED pattern obtained experimentally match the simulated SAED pattern of monoclinic GaTe (ICSD 8249) in Figure 1d. In addition, the XRD technique was also used to verify the crystal structure of GaTe. The XRD spectrum of a single-crystal GaTe piece in Figure 1e exhibits four prominent diffraction peaks corresponding to the (201), (402), (804), and (1005) planes, closely matching ICSD ID 8249, the same pattern used to verify the SAED results. The inset in Figure 1e illustrates the (201) direction using *VESTA*. For the quantitative and qualitative elemental characterization, EDX was carried out on an exfoliated GaTe flake on silicon, as shown in Figure 1f. The inset shows an SEM image of a GaTe flake, clearly showing its layered structure. The atomic composition percentages are 71.38% silicon, 14.45% gallium, and 14.17% tellurium, corresponding to a ratio of 50.49% Ga to 49.51% Te, which agrees with the ideal 50:50 stoichiometric ratio of GaTe. According to the EDX mapping in Figure 1g, the GaTe flake on silicon consists of gallium and tellurium signals in the same area, indicating a uniform stoichiometric compound. From the crystal and elemental analyses, it can be implied that the as-synthesized GaTe is purely monoclinic without oxide impurities.

### 3.2 Low-Temperature Raman Spectroscopy Study of GaTe

**Figure 2:**
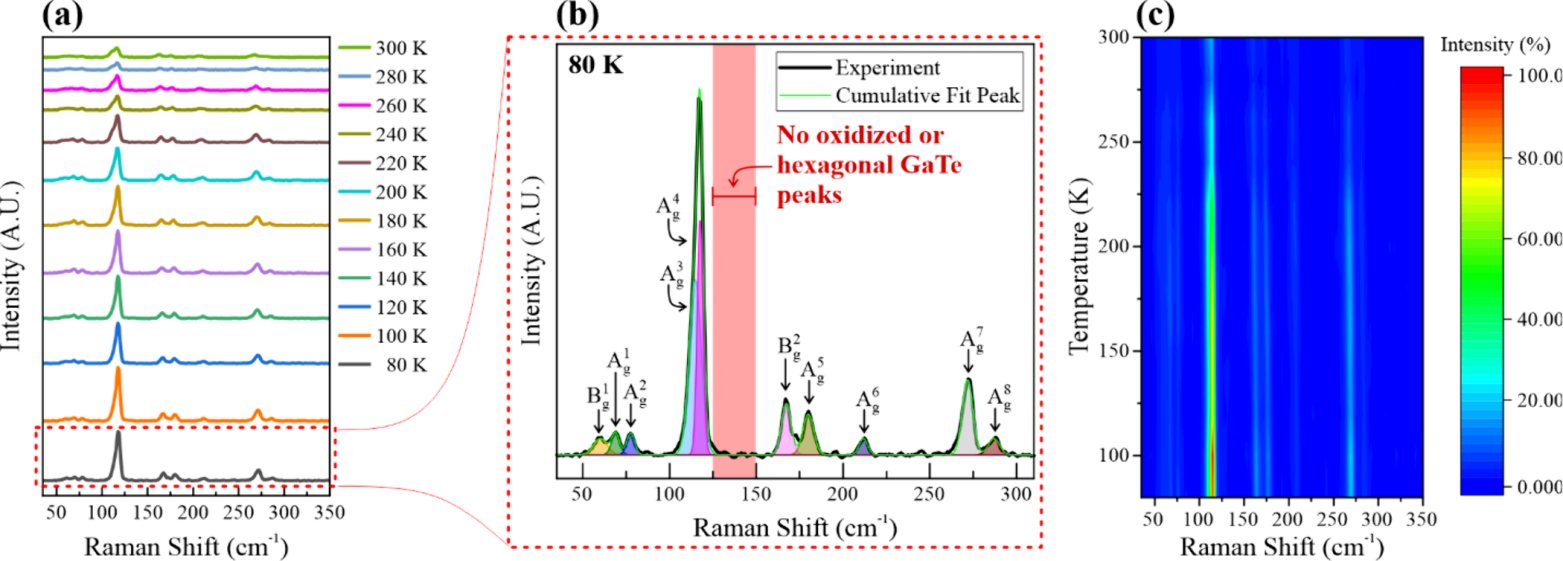
Raman spectroscopy of GaTe. (a) Temperature-dependent Raman spectra of a single-crystal GaTe flake obtained from 80 to 300 K. (b) Raman spectrum at 80 K showing the vibration modes of monoclinic GaTe. The region highlighted in red at 120–150 cm^–1^ shows no peaks related to hexagonal phase GaTe or oxidized GaTe. (c) Temperature-dependent Raman intensity mapping confirming that there is a slight peak position shifting primarily due to the thermal effect.

Raman spectroscopy is a versatile technique that can simultaneously identify the chemical composition and crystal phase of a material. Figure 2a displays the Raman spectra of a single-crystal GaTe flake from 300 to 80 K, showing 10 monoclinic GaTe peaks, 8 A_g_, and 2 B_g_ Raman vibration modes, which is consistent with other reports on monoclinic GaTe.^19,23,37^ The peaks are approximately located at 60, 69, 77, 114, 117, 167, 179, 211, 272, and 287 cm^–1^, as shown in the Raman spectrum at 80 K in Figure 2b. The highlighted part (∼120–150 cm^–1^) in the graph is the region where oxidized GaTe is commonly reported. In addition, hexagonal GaTe features are also reportedly observed at the same range.^18,46,47^ However, no such peaks are observed in our as-synthesized single-crystal GaTe, confirming that its composition consists purely of monoclinic GaTe. Figure 2c shows the corresponding intensity mapping of the temperature-dependent Raman spectra in Figure 2a. There is a slight shift in the Raman peaks as the temperature decreases. This is a common occurrence for any semiconducting materials mainly due to less intensity of the electron–lattice interaction affecting the scattered photons. The Raman vibration frequency is shifted to higher energies, and the intensity is enhanced at the same time.^48,49^ The peak position versus temperature plots of the single-crystal GaTe flake is shown in Figure S1. The temperature-induced Raman peak position shifting from 80 to 300 K can be fitted by the first-order linear equation as
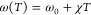
1where ω(*T*) is the Raman shift at a particular temperature *T*, ω_0_ is the Raman shift at 0 K by linear extrapolation, and χ is the first-order temperature coefficient or the slope of the linear fit.^48,49^ The peaks B_g_^1^ (55 cm^–1^), A_g_^1^ (63 cm^–1^), A_g_^2^ (75 cm^–1^), A_g_^3^ (111 cm^–1^), A_g_^4^ (114 cm^–1^), B_g_^2^ (162 cm^–1^), A_g_^5^ (174 cm^–1^), A_g_^6^ (205 cm^–1^), A_g_^7^ (267 cm^–1^), and A_g_^8^ (281 cm^–1^) have temperature coefficients of −0.0092, −0.0129, −0.0045, −0.0069, −0.0092, −0.0167, −0.0205, −0.0211, −0.0149, and −0.0202 cm^–1^/K, respectively. A previous paper reported that GaTe undergoes a phase transition at ∼261 K from hexagonal to monoclinic through calculations.^50^ However, there are no significant disappearances of any peaks or the emergence of new peaks in the Raman spectra, suggesting that the single-crystal GaTe flake does not undergo any phase transitions or degradation during the low-temperature Raman measurements.

### 3.3 Low-Temperature PL and PR Spectroscopy Studies of GaTe

**Figure 3:**
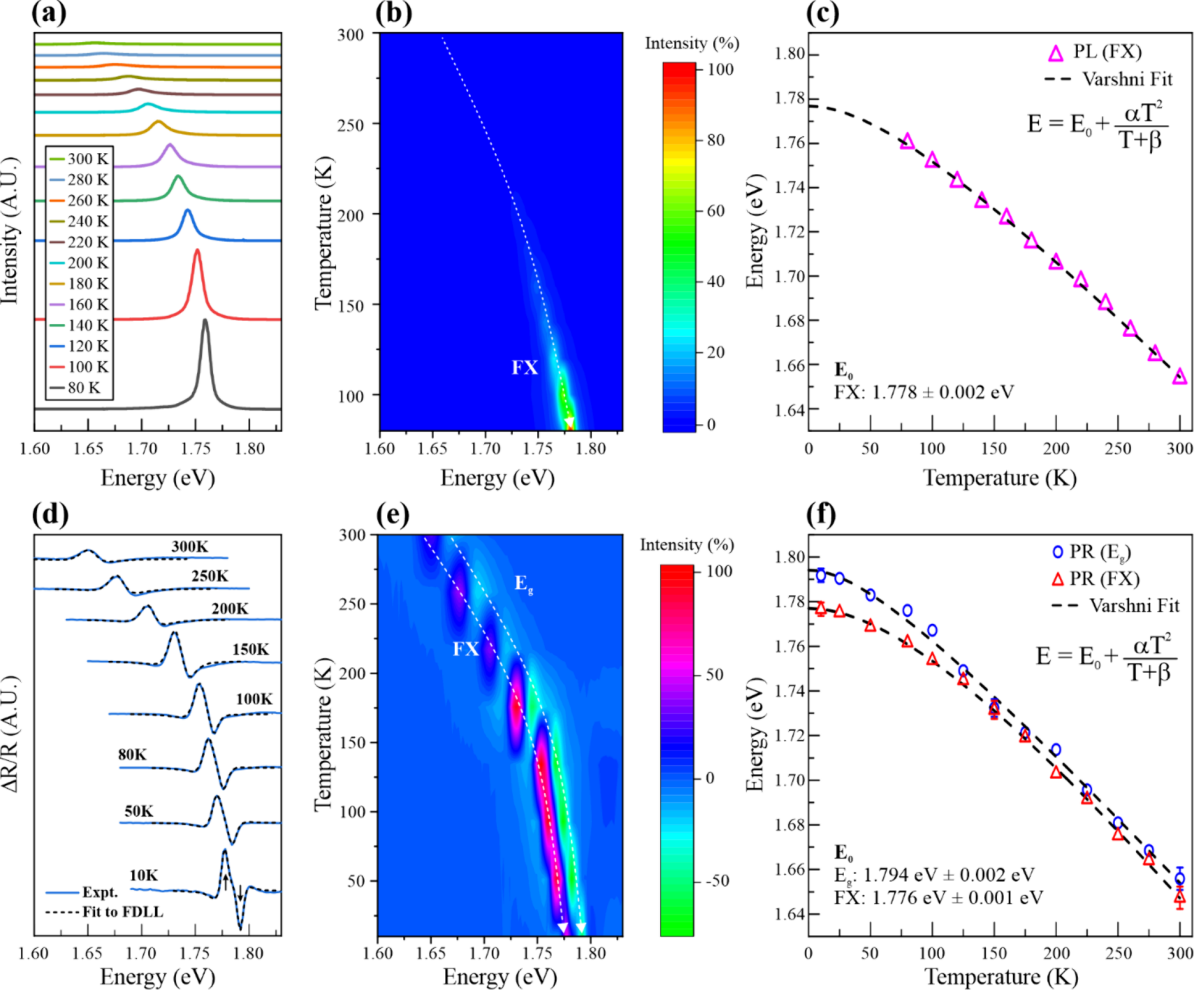
Temperature-dependent PL and PR spectra of GaTe. (a) Temperature-dependent PL spectra of GaTe from 80 to 300 K. The peak represents the FX emission.^22^ (b) Corresponding PL intensity mapping showing the FX emission shift. (c) Varshni fitting of the FX emission. (d) Temperature-dependent PR spectra of GaTe from 10 to 300 K. Each spectrum has two major upward and downward features, which represent the FX state and CB edge transitions.^60^ (e) Intensity mapping corresponding to the temperature-dependent PR spectra. The dashed lines indicate the energy positions of the upward and downward features, which embody the evolutions of the FX and CB edge transitions. (f) Two ideal Varshni fittings for the energy positions of the upward and downward features, confirming that the CB edge and FX transitions are present.

PL spectroscopy is used to study excitonic emissions for semiconductors. Figure 3a illustrates an evolution of excitonic emissions of bulk GaTe at lowered temperatures. The PL spectra have only one main PL peak located at ∼1.65 eV at room temperature, and the PL peak becomes sharper and is blueshifted at lowered temperatures. The evolution of the FX emission in semiconductors at lowered temperatures is normally associated with the effect of thermal suppression on phonons that can scatter excitons. Namely, electron–phonon scattering is minimal at lowered temperatures, so the energy of the excitonic emission is blueshifted to high energies and the excitonic intensity is enhanced.^51,52^ Moreover, no bound exciton emission is observed in the PL spectra. From the other previous studies, bound excitons in GaTe are normally observed in the PL spectra obtained at ∼150^22,24^ or ∼90 K.^23^ Also, there are no PL peaks related to the hexagonal phase GaTe, which has a prominent PL emission located at ∼1.588 eV and an estimated *E*_0_ of ∼1.73 eV.^24^ As per previous studies,^19,20,22,35,36^ monoclinic GaTe has a room-temperature FX emission located at 1.6–1.7 eV, so the PL peak represents an FX emission. Figure 3b demonstrates the corresponding PL intensity mapping for evolution of the FX emission. The PL peak’s blueshift can be fitted for the evolution of the FX emission at lowered temperatures using Varshni’s empirical equation
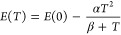
2where *E*(*T*) is the energy of a specific energy transition (or emission) feature at temperature *T*, *E*(0) is the energy at 0 K, and α and β are constants with units of electronvolts per Kelvin and Kelvin, respectively.^51^Figure 3c shows that the fitting is plotted as a function of the temperature. The thermal expansion of the crystal lattice has a minimal contribution to the FX emission at 0 K. The energy [*E*(0) =*E*_0_] is ∼1.778 eV for the FX emission at 0 K, which agrees with several previous reports^22,24^ for the FX emission of GaTe.

The electron transitions in bulk GaTe are further analyzed using temperature-dependent PR spectroscopy.^53,54^ The mechanism of PR spectroscopy allows one to observe not only excitonic emission but also optical absorption.^53,54^ The PR spectra in Figure 3d show an evolution of PR signals at lowered temperatures from room temperature to 10 K. Obviously, there are two features, an upward peak and a downward peak, located at ∼1.647 and ∼1.657 eV, respectively. The upward peak located at ∼1.647 eV represents the excitonic emission for FXs, bound excitons, trions, or trapping states.^23,55−59^ However, the downward peak located at ∼1.657 eV embodies an optical absorption for the band-gap energy (*E*_g_) of GaTe because the located energy of the downward peak is 10 meV larger than that of the upward peak. Both the upward and downward peaks are blueshifted in the evolution of the PR signals. The upward and downward peaks become sharper, and both PR intensities are higher at lowered temperatures. Figure 3e illustrates the corresponding PR intensity mapping for the evolution of excitonic emission and optical absorption. The PR peaks’ blueshifts can also be fitted for the evolution of the FX emission and *E*_g_ at lowered temperatures using Varshni’s empirical equation (eq 2). Figure 3f shows the two fittings plotted as a function of the temperature for the FX emission and *E*_g_. The energies of the FX emission and *E*_g_ are ∼1.776 and ∼1.794 eV at 0 K. The energy of the FX emission is 18 meV smaller than that of *E*_g_. The FX result of the PR evolution agrees with that of the PL evolution at lowered temperatures. Table 1 lists previous studies on various measurements of *E*_g_ and FX emission of bulk GaTe at 0 and 300 K, respectively. All of the FX emissions are similar at 0 and 300 K. However, only PR spectroscopy can show the results of both FX emissions and *E*_g_ in this study.

**Table 1:** Studies on *E*_g_ and FX Emission at 0 and 300 K

experiment	synthesis method	*E*_g_ at 300 K (eV)	*E*_g_ at 0 K (eV)	FX at 300 K *E* (eV)	FX at 0 K *E*_0_ (eV)	ref
PR	Bridgman	1.656	1.794	1.647	1.776	this work
PL	Bridgman	N.A.	N.A.	1.65	1.778	this work
PL/TR	CVT	N.A.	N.A.	1.652	1.778	(22)
PL/TR	CVT	N.A.	N.A.	1.652	1.786	(24)
PL	commercial purchase	N.A.	N.A.	1.65	1.759	(40)

**Figure 4:**
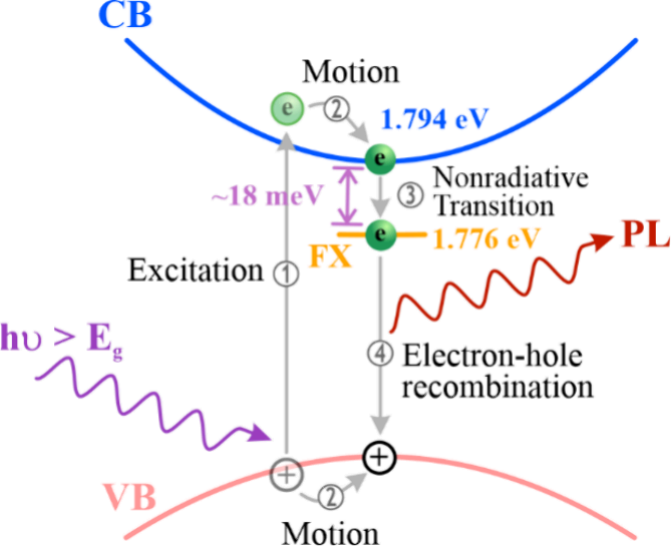
Absorption–emission mechanism. There are four steps for the absorption–emission mechanism. Step 1 is an electron excited from the VB to the CB by an incident light, and a hole is created at the VB. The excited electron is attracted by the created hole to form an FX due to Coulomb interaction. Note that the energy of the incident light is larger than *E*_g_. Step 2 is an FX motion. The excited electron freely moves to the lowest CB and the created hole moves to the highest VB simultaneously due to Coulomb interaction. Step 3 is a nonradiative transition. The excited electron falls into a virtual state (called the FX state) still due to Coulomb interaction. Step 4 is an electron–hole recombination. When the excited electron at the FX state drops into the created hole at the VB, an electron–hole recombination occurs and PL light is emitted.

According to previous studies^60−62^ and the factor that an optical emission is always accompanied by an optical absorption in semiconductors, a schematic diagram in Figure 4 demonstrates an absorption–emission mechanism with four steps, making a clear concept for optical absorptions and emissions of bulk GaTe. The optical absorption is an electron transition corresponding to an electron excited from the valence band (VB) to the CB by incident light. Note that the energy of the incident light is larger than *E*_g_. When an electron is excited to the CB, a hole is created at the VB. The excited electron is attracted by the created hole to form an exciton due to Coulomb interaction. The electron excitation is performed in step 1. Then the excited electron freely moves to the lowest CB, and the created hole moves to the highest VB simultaneously because of the Coulomb interaction. Namely, the exciton freely moves, so the exciton is a so-called FX. The FX motion is called step 2. Once the excited electron moves to the lowest CB, it falls into a virtual state (called the FX state), which is ∼18 meV underneath the lowest CB due again to Coulomb interaction. The created hole remains at the highest VB. The excited electron transition from the lowest CB to the FX state is a nonradiative transition, which is step 3. After the excited electron at the FX state drops into the created hole at the VB, electron–hole recombination occurs, and PL light is emitted. The electron–hole recombination is an excitonic emission, which is step 4. The absorption–emission mechanism shown in Figure 4 is similar to that reported in a previous study.^63^

### 3.4 Low-Temperature Photodetection of GaTe

**Figure 5:**
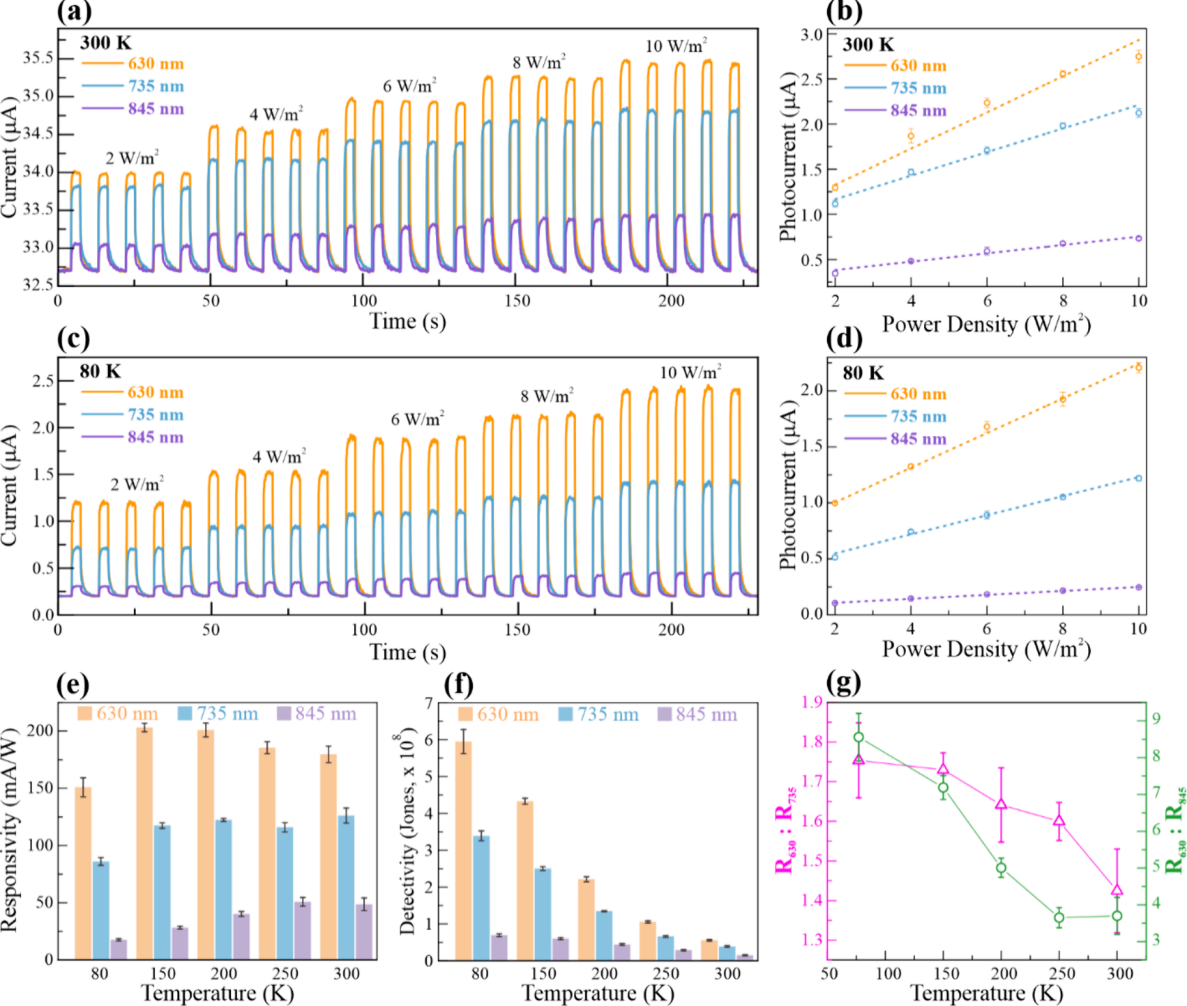
Temperature-dependent photodetection of GaTe. (a) Variable-power density photoresponse of GaTe. (b) Corresponding responsivity curve at 300 K. (c) Variable-power density photoresponse of GaTe. (d) Corresponding responsivity curve at 80 K. (e) Detectivity and (f) responsivity calculations at various temperatures. (g) Ratio of responsivity (and detectivity) at 630 nm to 735 and 845 nm, highlighting the effect of temperature-induced band-gap shifting.

Photodetection experiments at variable temperatures were carried out to study the effect of the temperature-induced band-gap shift. The ITO/glass-GaTe-ITO/glass sandwich configuration device was tested using 630, 735, and 845 nm light sources. Parts a and c of Figure 5 show the response of the single-crystal GaTe flake at 300 and 80 K, respectively, during irradiation and under dark conditions as a function of time. The current passing through the ITO/glass-GaTe-ITO/glass device increases when exposed to light, indicating photodetector behavior. In addition, the photocurrent is proportional to the incident power density, as shown in the responsivity curves at 300 and 80 K in parts b and d of Figure 5, respectively. The responsivity curve is obtained by measuring the photocurrent levels at various power densities: 2, 4, 6, 8, and 10 W/m^2^. The photodetector responsivity at a specific power density range is calculated using the equation
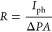
3where *R* is the responsivity, *I*_ph_ is the photocurrent, Δ*P* is the power density range (2–10 W/m^2^), and *A* is the photoactive sensing area.^64,65^ In this device, the photoactive material is the GaTe flake of about 1 × 1 mm in surface area. From the room temperature responsivity curve in Figure 5b, the ITO/glass-GaTe-ITO/glass photodetector exhibits the highest responsivity under exposure to 630 nm (180 ± 7.2 mA/W), followed by 735 nm (126 ± 6.5 mA/W) and 845 nm (49 ± 5.5 mA/W). Because the single-crystal GaTe flake has a measured band gap of ∼1.65 eV (∼747 nm), photons with less energy are not as highly absorbed compared with those above that energy. As a result, fewer electrons are converted to photocurrent. The same trend can be observed at 80 K, as shown in Figure 5d. Full photoresponse data taken at 80, 150, 200, 250, and 300 K and their corresponding responsivity curves can be found in Figure S2. Figure 5e shows the responsivity as a function of the temperature. As the temperature is reduced, the responsivity decreases. This effect is mainly due to the inverse relationship between the temperature and electrical resistance in semiconductors.^66,67^ However, the detectivity calculations in Figure 5f show an opposite response to the temperature. Detectivity is largely dependent on the dark current, which is suppressed at low temperatures, resulting in an enhancement.^68^ Detectivity is a photodetector parameter that measures its capability to distinguish changes in the photocurrent with respect to its dark current *I*_D_. It is calculated using the equation
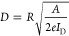
4where *D* is the detectivity, *R* is the responsivity, *A* is the photoactive sensing area, *e* is the electron charge, and *I*_D_ is the dark current.^64,65^ Overall, the performance of the ITO/glass-GaTe-ITO/glass photodetector at lower temperatures is enhanced. Although the responsivity decreased (−15.9%, −31.7%, and −63.7% for 630, 735, and 845 nm, respectively) with decreasing temperature, the detectivity is significantly improved (+972%, +800%, and +363% for 630, 735, and 845 nm, respectively) due to thermal suppression of the dark current. Moreover, the responsivity to lower-energy light exhibited a larger decrease than that to energy greater than the band gap. This suggests that the decrease in responsivity is also influenced by the shift in the band gap due to temperature.

In the study of low-temperature photodetection, there are two major factors affecting the performance: the temperature dependence of resistivity and the low-temperature-induced band-gap shifting to higher energy. The effect of band-gap shifting can be observed by comparing the photoresponse measurements in Figure 5a,c. With respect to the response at 630 nm exposure, the photocurrent level when exposed to 735 and 845 nm light decreases when the temperature is lowered to 80 K. For further investigation, the ratio of the responsivity at 630 nm (*R*_630_) to the responsivity at 735 nm (*R*_735_) and 845 nm (*R*_845_) is examined, as shown in Figure 5g. Because detectivity can be expressed as a function of the responsivity, taking their ratios would give the same value. Note that 630 nm is well above the band-gap energy, 735 nm is located around the band-gap energy (at a temperature range of 300–80 K), and 845 nm is significantly below the band gap. As the temperature is lowered from 300 to 80 K, the values of *R*_630_:*R*_735_ and *R*_630_:*R*_845_ decrease, indicating a relative decrease in responsivity to 735 and 845 nm light. Because the ratio is taken at the same temperatures, it can be implied that the relative decrease in responsivity is only due to the temperature-induced band-gap shifting. The photocurrent is generated as GaTe absorbs photons to excite the electrons from the VB to the CB, where it is free to conduct an electric current. The 735 nm (1.70 eV) light source is within the range of the band gap of GaTe during the low-temperature blueshift. From the PL and PR results, the band gap of the single-crystal GaTe flakes is ∼1.80 and ∼1.65 eV at 80 and 300 K, respectively. At 300 K, the incident light energy is above the band gap, but at 80 K, it is less than the band gap. As a result, photons are absorbed less as the temperature decreases, resulting in a smaller responsivity. The same effect occurs under exposure to 845 nm (∼1.47 eV), which is below the band gap of the single-crystal GaTe even at room temperature. The electrons excited to the CB by 845 nm light are further reduced as the band-gap shifts to higher energies at lower temperatures.

## 4 Conclusion

Monoclinic GaTe single crystals synthesized by the Bridgman technique were characterized by electron microscopy, diffractometry, EDX, and Raman, PL and PR spectroscopy, respectively. During the cooling/heating process, no structural transitions and oxidations were observed, implying that monoclinic GaTe single crystals are stable in ambient conditions. PL spectroscopy observed the FX emission of the GaTe single crystal, but PR spectroscopy detected not only the FX emission but also the CB edge absorption. The PL evolution at lowered temperatures can be fitted by Varshni’s equation for the GaTe single crystal, and the energy of the FX emission is obtained as ∼1.778 eV at 0 K. However, the PR evolution at lowered temperatures can be fitted by Varshni’s equation for the GaTe single crystal, and the two energies of the FX emission and band gap (*E*_g_) are acquired as ∼1.776 and ∼1.794 eV at 0 K. The band-gap blueshift at lowered temperatures influences the photoresponsivity of the GaTe photodetector at various wavelengths of incident light. As the temperature is lowered from 300 to 80 K, the photoresponsivity is decreased by −15.9%, −31.7%, and −63.7% for the wavelengths of 630, 735, and 845 nm, respectively.

## Supporting Information Available

Temperature-dependent Raman peak position, full photoresponse data (300, 250, 200, 150, and 80 K), and PR spectrum of a few-layer GaTe flake (PDF)
