# Supplementary material for: Temperature Dependence of the Band Gap and Exciton Photoreflectance in Layered Gallium Telluride
Source: ACS Appl Mater Interfaces. 2025 Feb 3;17(6):9514–22. doi: 10.1021/acsami.4c17178 (PMC11826511; doi:10.1021/acsami.4c17178)
Supplement: Supplementary file 1 — am4c17178_si_001.pdf [file am4c17178_si_001.pdf]

Supporting Information

Temperature dependence of bandgap and exciton photoreflectance in layered gallium telluride

*Carlo C. Sta. Maria[1,†], Po-Hung Wu[2,†], Denny Pratama Hasibuan[1], Clara Sinta Saragih[1], Hien Giap[1], Duc Huy Nguyen[1], Yan-Ruei Chen[4], Giang Thi Phan[1], Duy Van Pham[1] Ji-Lin Shen[3], Chien-Chih Lai[1], Maw-Kuen Wu[4], and Yuan-Ron Ma[1,5]**

[1]Department of Physics, National Dong Hwa University, Hualien 97401, Taiwan

[2]Department of Electrical Engineering, National Dong Hwa University, Hualien 97401, Taiwan

[3]Department of Physics, Chung Yuan Christian University, Taoyuan 32023, Taiwan

[4]Institute of Physics, Academia Sinica, Taipei 11529, Taiwan

[5]Office of Postgraduate Studies, UCSI University, Kuala Lumpur 56000, Malaysia

*Correspondence: ronma@gms.ndhu.edu.tw

†These authors contributed equally.

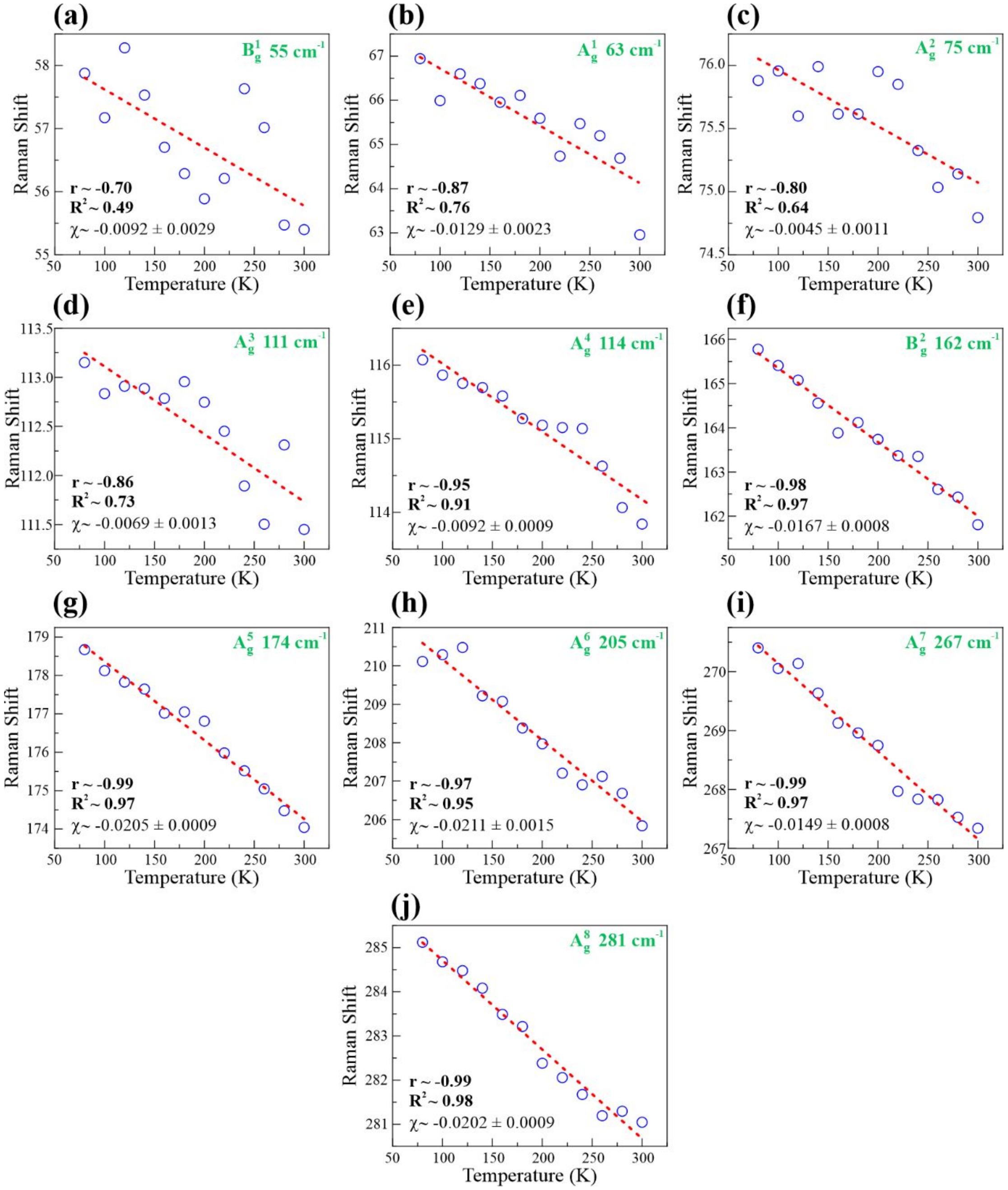

Figure S1. Temperature-dependence Raman blueshifts. Graphs of Raman shifts versus temperatures show linear blueshifts at lowered temperatures for 10 various A_g and B_g Raman modes, including (a) $B_g{}^1$, (b) $A_g{}^1$, (c) $A_g{}^2$, (d) $A_g{}^3$, (e) $A_g{}^4$, (f) $B_g{}^2$, (g) $A_g{}^5$, (h) $A_g{}^6$, (i) $A_g{}^7$ and (j) $A_g{}^8$, respectively. Note that the room-temperature Raman scatterings of the 10 various A_g and B_g Raman modes are located at 55, 63, 75, 111, 114, 162, 174, 205, 267 and 281 cm^{-1}, respectively.

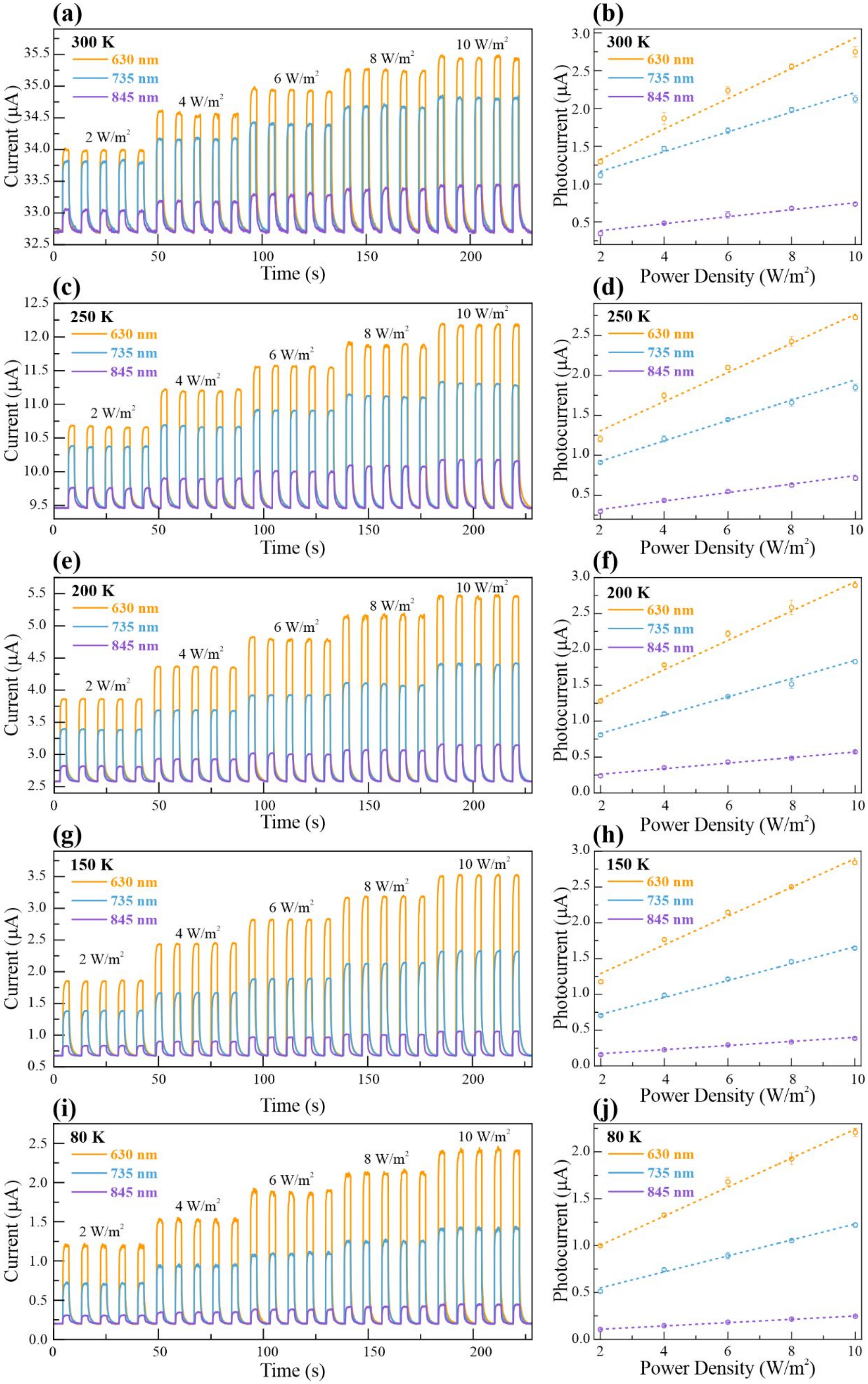

(a)
300 K
630 nm
735 nm
845 nm
2 W/m²
4 W/m²
6 W/m²
8 W/m²
10 W/m²
Current (μA)
Time (s)
(b)
300 K
630 nm
735 nm
845 nm
Photocurrent (μA)
Power Density (W/m²)
(c)
250 K
630 nm
735 nm
845 nm
2 W/m²
4 W/m²
6 W/m²
8 W/m²
10 W/m²
Current (μA)
Time (s)
(d)
250 K
630 nm
735 nm
845 nm
Photocurrent (μA)
Power Density (W/m²)
(e)
200 K
630 nm
735 nm
845 nm
2 W/m²
4 W/m²
6 W/m²
8 W/m²
10 W/m²
Current (μA)
Time (s)
(f)
200 K
630 nm
735 nm
845 nm
Photocurrent (μA)
Power Density (W/m²)
(g)
150 K
630 nm
735 nm
845 nm
2 W/m²
4 W/m²
6 W/m²
8 W/m²
10 W/m²
Current (μA)
Time (s)
(h)
150 K
630 nm
735 nm
845 nm
Photocurrent (μA)
Power Density (W/m²)
(i)
80 K
630 nm
735 nm
845 nm
2 W/m²
4 W/m²
6 W/m²
8 W/m²
10 W/m²
Current (μA)
Time (s)
(j)
80 K
630 nm
735 nm
845 nm
Photocurrent (μA)
Power Density (W/m²)

Figure S2. Photoresponses. (a) Various power-density and varying light-wavelength photoresponses measured at 300 K. (b) Various power-density photocurrents at 300 K. (c) Various power-density and varying light-wavelength photoresponses measured at 250 K. (d) Various power-density photocurrents at 250 K. (e) Various power-density and varying light-wavelength photoresponses measured at 200 K. (f) Various power-density photocurrents at 200 K. (g) Various power-density and varying light-wavelength photoresponses measured at 150 K. (h) Various power-density photocurrents at 150 K. (i) Various power-density and varying light-wavelength photoresponses measured at 80 K. (j) Various power-density photocurrents at 80 K. Note that three light-emitting-diodes (LEDs) with light wavelengths of 630, 735 and 845 nm are used for photoresponses, and they were applied in the power densities of 2, 4, 6, 8, 10 W/m^2, respectively.

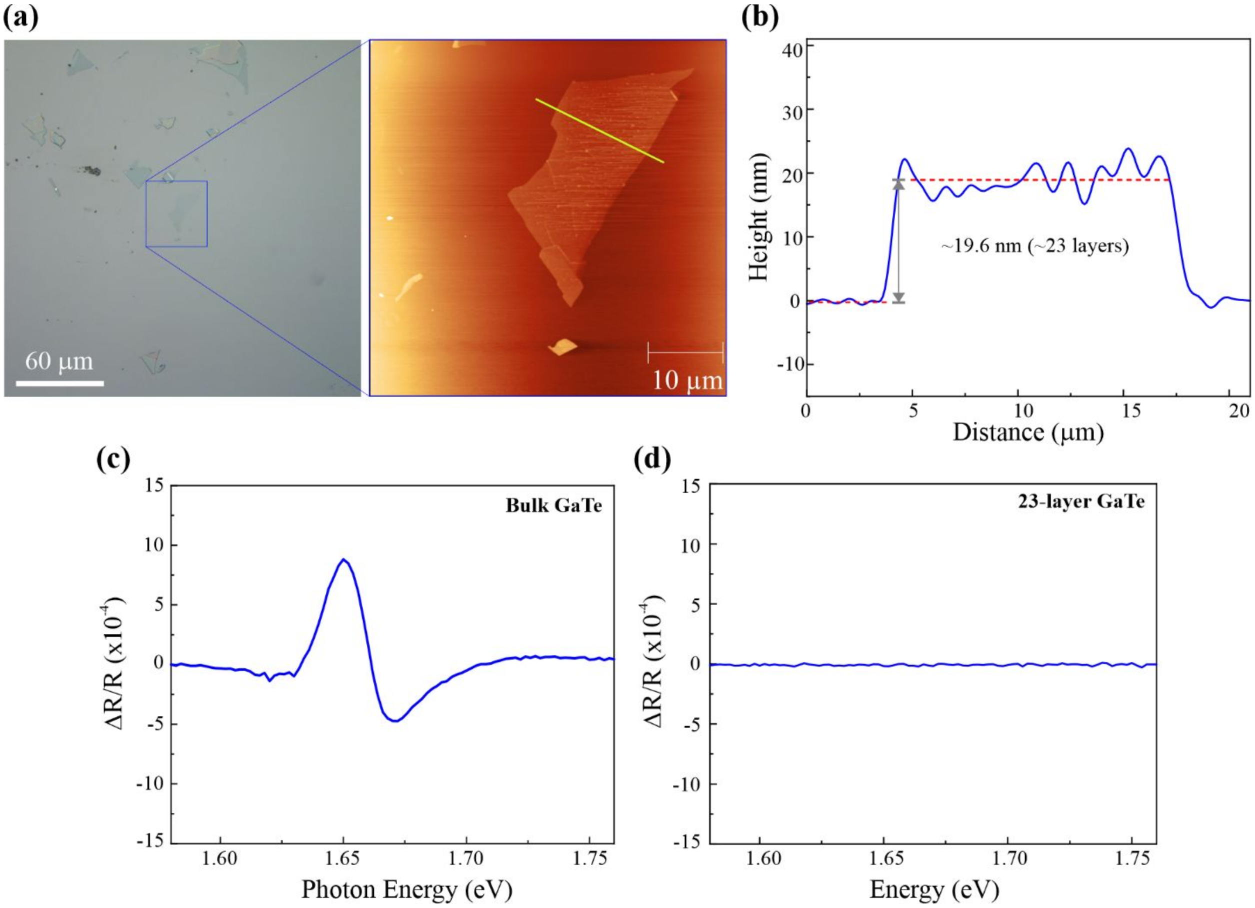

Figure S3. **Morphology, thickness and PR spectrum of a few-layer GaTe**. (a) OM (left panel) and AFM (right panel) images of a few-layer GaTe on a Si substrate. (b) AFM profile shows that the thickness of the few-layer GaTe is around 19.6 nm, so the few-layer GaTe has 23 layers. (c) Room-temperature PR spectrum of bulk GaTe has a strong upward peak located at ~1.65 eV. (d) Room-temperature PR spectrum of the 23-layer GaTe has no peak at all, indicating that one monolayer GaTe also has no PR signal (or absorption) at all.
